# Case Report: Reactive T-cell large granular lymphocytes in the bone marrow of a patient with relapsed/refractory multiple myeloma receiving teclistamab

**DOI:** 10.3389/fonc.2025.1630929

**Published:** 2025-08-06

**Authors:** Erin A. Dean, Li-Jun Yang, Robert P. Seifert, John R. Wingard

**Affiliations:** ^1^ Department of Medicine, Division of Hematology and Oncology, University of Florida, Gainesville, FL, United States; ^2^ Department of Pathology, Immunology, and Laboratory Medicine, University of Florida, Gainesville, FL, United States

**Keywords:** multiple myeloma, T-LGL, teclistamab, bispecific antibodies (BsAbs), bone marrow microenvironment (BMME)

## Abstract

T-cell engager therapies for the management of relapsed/refractory (R/R) multiple myeloma (MM) may lead to neutropenia with or without associated infections, which can limit treatment and efficacy. We present a case of a patient with penta-class R/R MM who, while receiving teclistamab, developed persistent severe to moderate neutropenia with associated infections. A review of her bone marrow confirmed the eradication of the malignant plasma cells, but flow cytometry identified an increase in T-cell large granular lymphocytes (T-LGLs). Upon further analysis, T-LGLs were found to be reactive and non-clonal, ruling out secondary T-LGL leukemia. This case describes the evolving peripheral blood cell counts, bone marrow composition, and radiologic findings due to the activation of the patient’s immune system before, during, and after treatment with teclistamab. To our knowledge, this is the first reported potential association of T-LGL activation with teclistamab treatment.

## Introduction

Multiple myeloma (MM) is an incurable plasma cell neoplasm typically managed in transplant-eligible patients with a quadruplet regimen followed by autologous hematopoietic stem cell transplant (HSCT) and maintenance therapy in the first-line setting and, in case of prolonged response to initial treatment, with a triplet regimen followed by second autologous HSCT/maintenance therapy in the relapsed setting (1). Due to nonfunctional immunoglobulin production because of MM and the immunosuppressive properties of the multidrug regimens, which may consist of chemotherapy, immunomodulatory drugs, proteosome inhibitors, and monoclonal antibodies, all patients are at risk of neutropenia and infections (1). Bispecific antibodies constitute a new class of drugs against MM. Teclistamab, an anti-B-cell maturation antigen (BCMA) and anti-CD3 commercially available bispecific antibody, can be used for the treatment of relapsed/refractory (R/R) MM following at least four prior lines of therapy (2). Neutropenia is a known common side effect that occurred in 70.9% of patients on the MajesTEC-1 trial, with grade 3 or 4 neutropenia in 64.2% of the cases (3). Given the high risk of infection due to neutropenia, understanding the mechanism behind this hematologic adverse event is crucial for the patients’ safety. Here we highlight the bone marrow findings of a patient who developed neutropenia while undergoing treatment with teclistamab for R/R MM.

## Case

**Figure 1:**
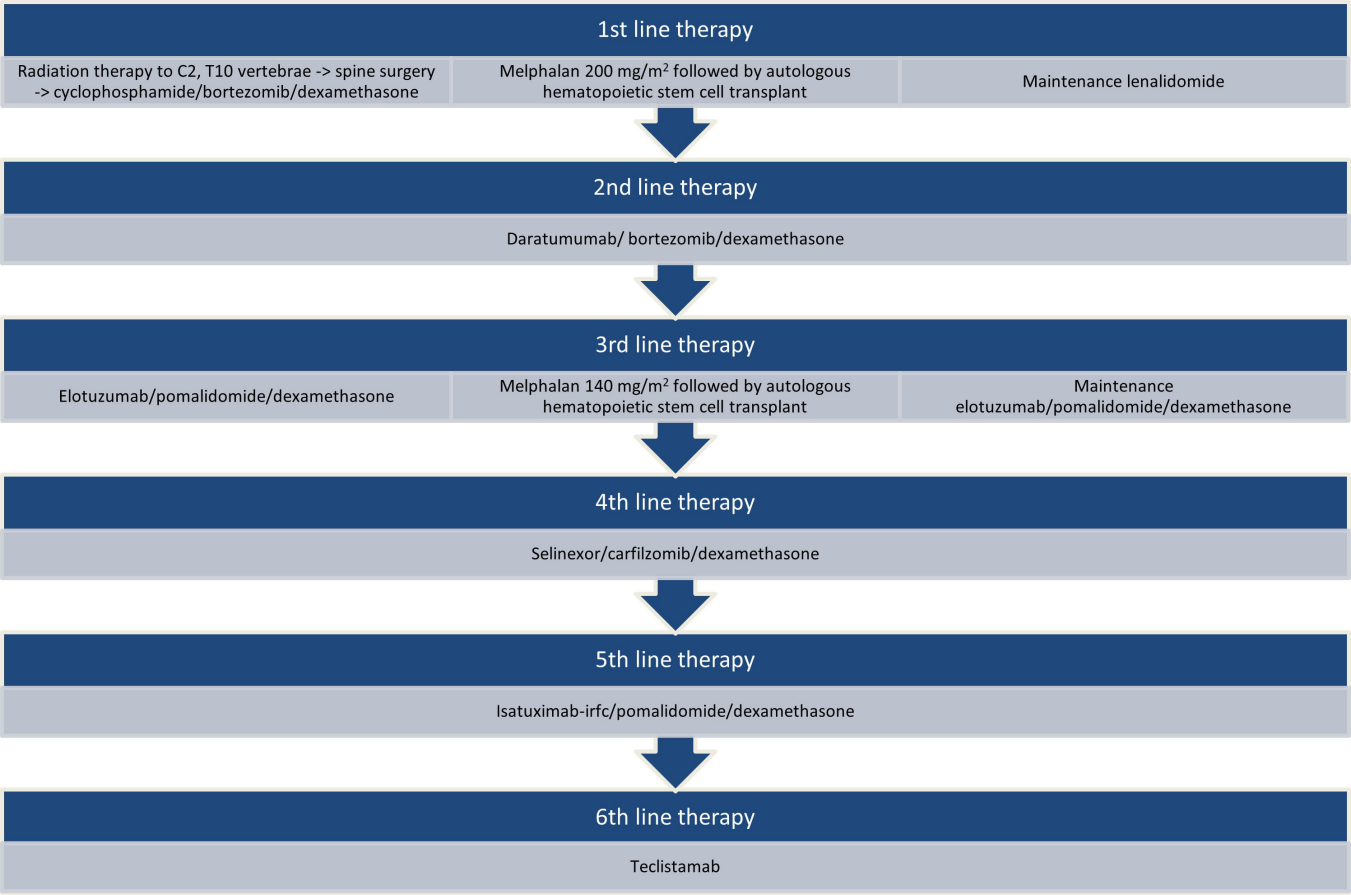
Lines of therapy prior to teclistamab. Sequencing of therapies received by the patient since the time of diagnosis to initiation of teclistamab.

The patient is an African-American, non-Hispanic woman in her early 70s with a history of obesity, hypertension, hyperlipidemia, type 2 diabetes mellitus, and gastroesophageal reflux disease managed for R/R Stage IIIA lambda light chain (LLC) MM with high-risk cytogenetics of t(11;14), 17P/TP53 deletion, and gains of chromosome 9 and 15, complicated by stage 4 chronic renal failure. She was diagnosed with MM in 2015 and received six lines of therapy in total (**Figure 1**). As part of the first line of therapy, the patient underwent localized radiation therapy to the myeloma lesions in her cervical (C) vertebrae 2 and thoracic vertebra (T) 10 followed by posterolateral arthrodesis of T8 through T12 and laminectomy with tumor debulking at T10; she then received 4 cycles of cyclophosphamide/bortezomib/dexamethasone, achieving a partial response; afterward, she was treated with melphalan (200 mg/m^2^) followed by autologous HSCT and lenalidomide maintenance therapy. She achieved a complete response. Maintenance therapy was discontinued 4 years later due to persistent anemia. In 8 months, the patient experienced her first disease relapse, for which she received 3 cycles of daratumumab/bortezomib/dexamethasone with inadequate response. Her therapy was changed to elotuzumab/pomalidomide/dexamethasone for 2 cycles before proceeding to melphalan (140 mg/m^2^), followed by autologous HSCT with elotuzumab/pomalidomide/dexamethasone post-transplant for 1 year and 3 months until progression of the disease. She had partial response to salvage autologous HSCT. Next, she received selinexor/carfilzomib/dexamethasone for 1.5 years. Upon further disease progression, she was switched to isatuximab-irfc/pomalidomide/dexamethasone for 6 months.

**Figure 2:**
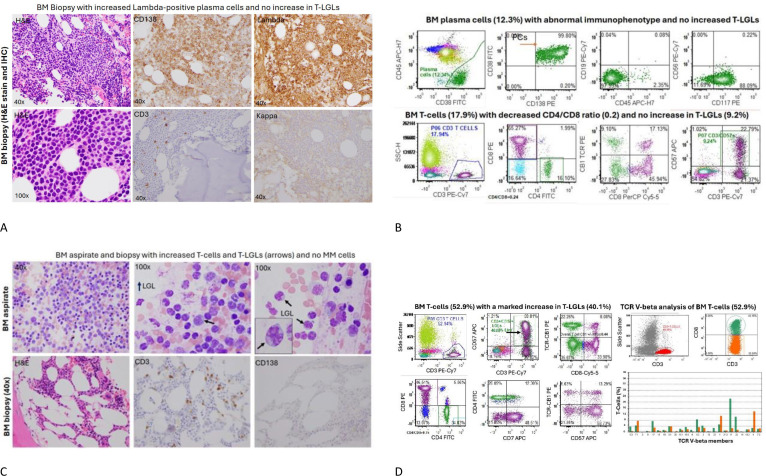
Bone marrow aspiration and biopsy at the start of and 12 months from the start of teclistamab. **(A)** The bone marrow sections showed hypercellular marrow with increased CD138+, lambda-restricted plasma cells, and rare scattered CD3+ T-cells and no kappa+ plasma cells present. ​**(B)** The concurrent bone marrow flow cytometry identified an abnormal plasma cell population (12.3%) with aberrant immunophenotype. The total bone marrow T-cells (17.9%), particularly CD3+/CD57+ T-LGLs (9.24%), were not increased, but there were relatively increased CD8+ T-cells with a decreased CD4/CD8 ratio (0.24). ​**(C)** After 12 months of teclistamab treatment, the patient had persistent neutropenia, with low cellularity and no abnormal plasma cells but with markedly increased CD3+ T-cells, particularly large granular lymphocytes with cytoplasmic granules (see the arrows).​ **(D)** Left panel: A corresponding bone marrow flow cytometric analysis revealed increased CD3+ T-cells (52.9%) and significantly increased CD3+/CD57+ T-large granular lymphocytes (T-LGLs) (40% of total cells). A pattern of T-cell receptor-constant β chain-1 (TCR-CB1) expression is a useful tool to detect T-cell clonality in the setting of T-large granular lymphocytic leukemia and other T-cell disorders. An abnormal pattern of TCR-CB1 expression was noted in CD8+ T-cells with a large subset of CD8+/CD57+ T-LGLs showing no expression of TCR-CB1, a sign suggestive of clonal expansion. Right panel: A TCR V-beta repertoire analysis by flow cytometry was performed to determine T-cell clonality using Beckman testing kit. As shown, CD8+ T-cells (green) and CD4+ T-cells (red) were plotted using antibodies against 24 TCR V-beta family members. One dominant CD8+ clone was noted (20% of CD8+ T-cells), and the rest of the T-cell clones were within oligoclonal range, with no definitive monoclonal expansion of cytotoxic T-cells. BM, bone marrow; H&E, hematoxylin and eosin; IHC, immunohistochemistry.

At the time of the latest progression of her disease, her serum myeloma markers were worsening with low immunoglobulins (Ig), elevated LLC at 782.62 mg/dL and low kappa/lambda (K/L) ratio of 0.00, and monoclonal protein (M-spike) 0.19 g/dL with immunofixation (IFE), showing the presence of free LLC monoclonal protein with an additional band in IgG kappa. The white blood cell count (WBC) was 2.1 × 10^3^/uL, absolute neutrophil count (ANC) 1.24 × 10^3^/uL, hemoglobin (Hgb) 7.4 g/dL, platelet count (Plt) 25 × 10^3^/uL, creatinine (Cr) 1.76 mg/dL (at baseline), and calcium (Ca) 7.9 mg/dL. The bone marrow aspirate showed a hypercellular marrow, over 50% cellularity, with 70% CD138 (+) lambda light chain restricted plasma cells. The flow cytometric analysis detected abnormal plasma cells with lots of CD19 and CD45 expression with dim CD117 and no CD56 expression (**Figures 2A, B**). The immunohistochemical testing for BCMA of plasmacytoid cells was variably positive (very weak cytoplasmic and membranous staining); 46, XX (8). The fluorescence *in situ* hybridization (FISH) for MM was positive for IGH/CCND1 gene loci fusion rearrangement and TP53 gene locus copy number loss but negative for chromosome 13q copy number loss and 1q21 loci copy number loss. A whole-body F-18-FDG positron emission tomography/computed tomography (PET/CT) scan demonstrated new diffuse FDG uptake in the entire axial skeleton and the proximal appendicular skeleton in sites of normal and abnormal bone consistent with disease progression (**Table 1**).

**Table 1:** Restaging serum, bone marrow, and imaging findings following teclistamab initiation and discontinuation.

Characteristic/time	Teclistamab initiation	9 months post-initiation	12 months post-initiation	3 months post-discontinuation
WBC (4.0–10.0 × 10^3^/uL)	2.1	3.8	3.5	5.4
ANC (1.70–7.00 × 10^3^/uL)	1.24	0.15	0.53	1.94
ALC (1.00–3.20 × 10^3^/uL)	0.63	1.56	2.00	2.59
Hgb (12.0–16.0 g/dL)	7.4	10.3	11.3	11.7
Plt (150–450 × 10^3^/uL)	25	146	115	103
Cr (0.38–1.02 mg/dL)	1.76	1.86	1.64	1.79
Ca (8.4–10.2 mg/dL)	7.9	10.0	9.1	9.6
IgG (635–1,616 mg/dL)	166	335	433	525
IgA (70–433 mg/dL)	<10	<10	<10	<10
IgM (45–281 mg/dL)	<20	<20	<20	<20
Serum KLC (0.33–1.94 mg/dL)	0.10	<0.06	<0.06	<0.06
Serum LLC (0.57–2.63 mg/dL)	782.62	<0.14	<0.14	<0.13
K/L ratio (0.26–1.65)	0.00	Unable to calculate	Unable to calculate	Unable to calculate
M-spike, IFE (none)	0.19 +LLC, +IgG kappa	0, none	0, none	0, none
Cellularity of the marrow (%)	> 50	30-40	10-30	30
Overt myeloma plasma cells in the marrow (%)	70	0	0	0
Polyclonal T-LGLs in the marrow (%)	0	0	55	0
Target lytic lesion in left sacral area on PET/CT, SUVmax (all listed values above normal uptake per reading radiologist)	9.5	3.24	5.11	4.7

ALC, absolute lymphocyte count; PET/CT, positron emission tomography/computed tomography; SUVmax, maximum standardized uptake value.

It should be noted that 24-h urine protein/urine myeloma studies are not available.

The patient was started as inpatient on treatment with teclistamab on the standard step-up dosing of 0.06 mg/kg on day 1, 0.3 mg/kg on day 4, and 1.5 mg/kg on day 7. Drug loading was complicated by cytokine release syndrome (CRS) maximum grade 2, which resolved with dexamethasone as per the institutional standard of care. Teclistamab was then continued outpatient at 1.5 mg/kg weekly. The treatment was interrupted due to infections, including four bacterial urinary tract infections requiring antibiotics and one upper respiratory infection (non-CoViD-19 coronavirus) requiring supportive care, as well as by scheduled bilateral cataract surgeries. As early as 3 months into the treatment, the patient started becoming severely immunocompromised with severe (ANC < 0.5 × 10^3^/uL) or moderate (ANC 0.5–1.0 × 10^3^/uL) neutropenia, due to which the doses of the bispecific drug were held and filgrastim was administered, and with hypogammaglobulinemia, for which she received intravenous immune globulin as per our clinic standard for IgG <600 mg/dL. During the entire treatment period, the drug had to be reloaded once after it was held for more than 28 days.

At 9 months after starting teclistamab, the serum myeloma markers were low or normal (**Table 1**). Due to the development of recurrent, persistent neutropenia, a restaging bone marrow biopsy was performed to evaluate for relapsing MM versus therapy-related myelodysplastic syndrome or acute myeloid leukemia versus another etiology and that showed a normocellular marrow, 30%–40%, with trilineage hematopoiesis with erythroid hyperplasia and no overt evidence of residual plasma cell neoplasm or other hematologic malignancy; normal female karyotype 46, XX [20]; FISH for MM that was negative for clonal aberrations; and next-generation sequencing panel (NGS) panel that showed variants of unknown significance only but no pathogenic or likely pathogenic variants. A whole-body PET/CT scan showed decreased FDG avidity of preexisting lytic pelvic lesions and throughout the spine. Given the positive treatment response at that point, the teclistamab administration frequency was changed to every 2 weeks.

After 12 months of therapy, the serum myeloma markers were consistent with continued controlled disease (**Table 1**), and restaging imaging was repeated to reevaluate the remaining active lytic lesions, which remained non-painful. The whole-body PET/CT, however, had increased FDG uptake of the lytic pelvic lesions and spine, which was concerning for progressive disease. The patient had no rheumatologic conditions, recent history of trauma, new pain anywhere, or clinical signs and symptoms to suggest acute or chronic infection/inflammation. She continued to have neutropenia with ANC < 1.0 × 10^3^/uL. A bone marrow aspiration and biopsy was therefore repeated, and it showed a hypocellular marrow, 10%–30%, for age with decreased trilineage hematopoiesis and relative erythroid hyperplasia, no overt evidence of residual plasma cell neoplasm, but with a mild increase in reactive T-cells, 55% of total cells, including CD3+/CD8+/CD57+ T-cell large granular lymphocytes with atypical pattern of TCR-CB1 expression and with T-cell receptor V-beta repertoire analysis showing polyclonal T-cells with two small oligoclones noted in CD8+ T-cells (V-beta 11 at 20% of CD8+ T-cells and V-beta 22 at 12.7%), but no overt evidence of monoclonal T-cell expansion (**Figures 2C, D**); 46, XX [20]; FISH for MM remained negative; and the NGS panel showed variants of unknown significance only but no pathogenic or likely pathogenic variants.

**Figure 3:**
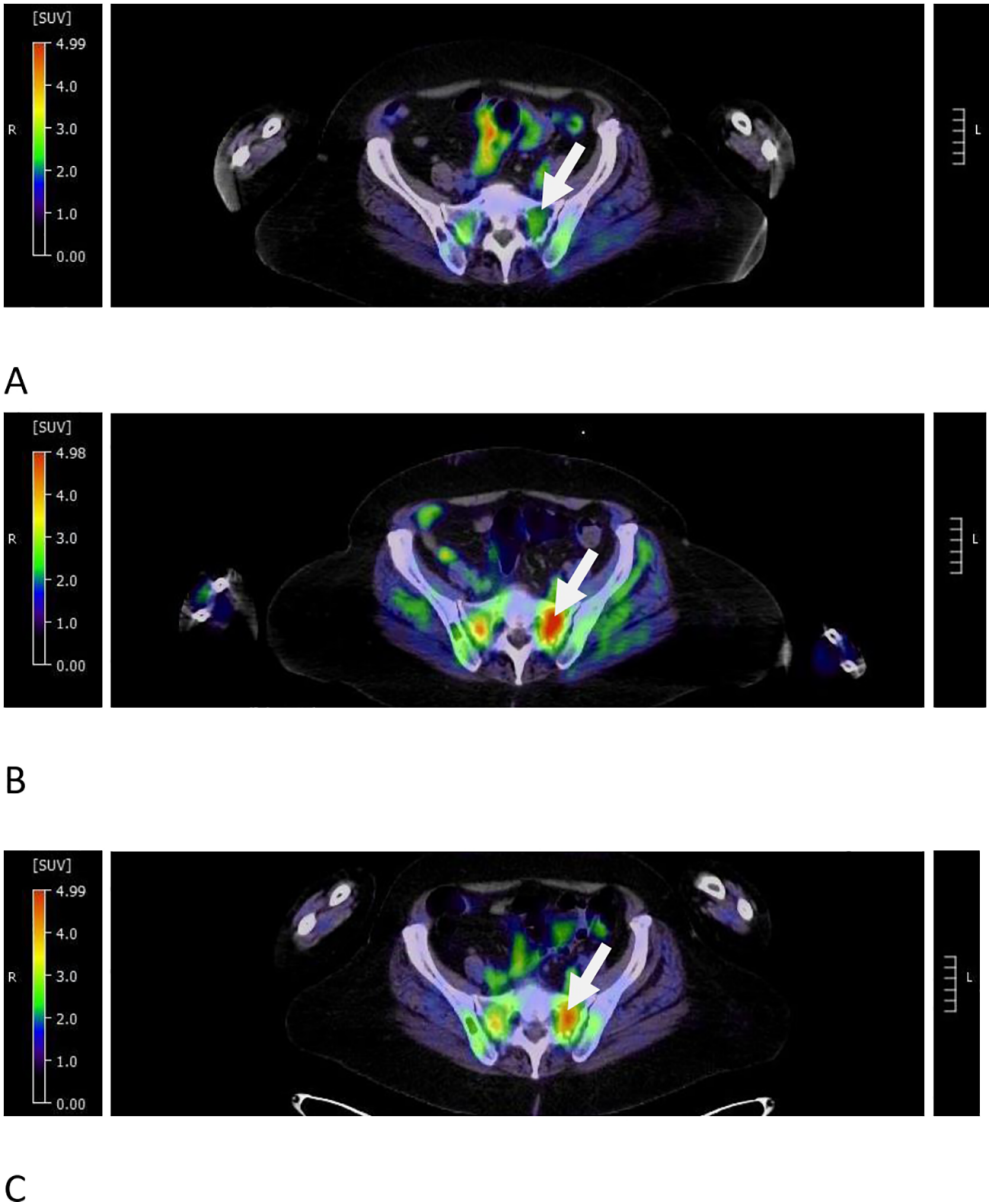
Pseudoprogression in target FDG avid pelvic lesion during teclistamab treatment. **(A)** At 9 months from the start of therapy, the left sacral lesion had a decreased SUVmax of 3.24 from 9.5 at relapse of the disease (see the arrow). **(B)** At 12 months from the start of therapy, the same dominant lesion demonstrated increased SUVmax to 5.11 (see the arrow). **(C)** At 3 months after the discontinuation of therapy, the SUVmax showed a minimal interval decrease to 4.7 (see the arrow).

After a discussion with the patient, teclistamab was discontinued to try to allow neutrophil recovery, and she was monitored off therapy with monthly serum myeloma studies. In 3 months, the neutrophil count normalized and a repeat bone marrow biopsy showed a normocellular marrow, 30% cellularity, with trilineage hematopoiesis with no overt evidence of plasma cell neoplasm; 46, XX [20]; FISH for MM was negative; and no minimal/measurable residual sequences were detected by NGS (limit of detection 10^6^). A whole-body PET/CT demonstrated a mild decrease in FDG uptake of the lytic lesions in the pelvis (**Figure 3**) and spine without evidence of disease progression. At the time of this report, the patient continued to be under close observation while off therapy.

## Discussion

Our patient’s hematologic tolerance of the BCMAxCD3-directed bispecific antibody teclistamab was similar to that of the patients in the MajesTEC-1 trial and real-world analyses whose treatment was complicated by neutropenia and infections. In the phase 1–2 study of teclistamab, infection occurred in 76.4% of the patients, and granulocyte colony-stimulating factor (G-CSF) was administered to 91 out of the 117 patients for neutropenia (3). In a German study of 123 patients, 54.5% had infection (4). In another real-world analysis of 106 patients with R/R MM who received the drug, infections and grades 3 to 4 neutropenia still occurred in 31% and 21% of patients, respectively (5). Our patient was penta-class refractory and immunocompromised due to the heavy prior treatment at the start of therapy. She experienced recurrent hypogammaglobulinemia requiring IVIG, infections, and persistent neutropenia requiring G-CSF without development of fever or need for hospitalization.

A repeat bone marrow sampling at 12 months post-treatment, performed to evaluate the patient’s persistent neutropenia, showed an increase in reactive T-cells compared to bone marrow sampling at 9 months. Despite these being identified as CD3+/CD57+ T-cell large granular lymphocytes (T-LGLs), on further characterization of the T-cells, there was no monoclonal expansion of cytotoxic CD8+ T-cells to support a diagnosis of T-LGL leukemia. Two oligoclonal populations were detected instead, that is, two quantitatively small CD8(+) T-cell populations each expressing a different TCR V-beta epitope. Such findings are not diagnostic for T-cell leukemia as they can be commonly seen in auto-immune and drug-mediated conditions. T-cell leukemias, in contrast, typically demonstrate a single dominant population restricted for a given TCR V-beta. The patient’s pre-treatment bone marrow flow cytometric analysis from 15 months earlier showed no significant increase in CD8(+)/CD56(+) T-LGLs (8.99%), and they demonstrated a polyclonal expression of TCR-CB1. The finding of oligoclonal expansion by TCR V-beta repertoire analysis in the post-treatment specimen is more consistent with an immune response than a pre-neoplastic finding. While clinically 50% of patients with T-LGL leukemia have neutropenia, with 20% as severe neutropenia and 15%–39% as infections, they also display peripheral lymphocytosis, which our patient did not, and their bone marrow is typically hypercellular, not hypocellular like in this case, with usually less than 50% infiltration by CD3+, CD8+, and CD57+ clonal LGL cells (6).

Upon engagement of T-cells, bispecific antibodies like teclistamab may trigger dysregulation of the immune system. A known result of this is the release of measurable inflammatory cytokines, such as interferon-γ, interleukin-6, interleukin-10, and interleukin-2 receptor α (3), predominantly during the step-up dosing or after the first full dose of the drug. Clinical manifestations include CRS and immune effector cell-associated neurotoxicity syndrome, which, in the MajesTEC-1 trial, occurred in 72% and 3% of patients, respectively (3). Additionally, both peripheral and bone marrow microenvironment changes of the immune system can occur in patients receiving T-cell engager therapies. In the correlation analysis of immune fitness with response to therapy in the MajesTEC-1 trial, at pre-treatment, the clinical responders were found to have a higher circulating number of naïve CD8+ T-cells and bone marrow with more cytolytic potential and CD8+ T-cells expressing granzyme B and perforin (7). In a separate study of 16 patients’ pre- and post-teclistamab bone marrow, CD8+ effector T-cells underwent expansion in those with response (8). Similarly, in our responding patient, an increase in CD8+ T-cells was observed with a dominant clone corresponding to 20% of all CD8+ T-cells. Uniquely, a year into treatment, there was a large new population of T-LGLs CD8+/CD57+ and CD3+/CD57+ that were not the result of monoclonal expansion but likely reactive in the setting of teclistamab. We hypothesized that these reactive T-LGLs suppressed the hematopoiesis of neutrophils, and 3 months after therapy discontinuation, the bone marrow cellularity and hematopoiesis were restored to normal. The patient became infection-free.

T-LGL clonal expansion has been observed in association with various conditions as well as treatments and has potentially been linked to otherwise unexplained cytopenias. Viral infections, immune thrombocytopenia, splenectomy, non-Hodgkin lymphoma, solid tumors, and hemophagocytic syndrome have all been associated with the appearance of reactive T-LGLs (9). In a retrospective study of 150 engrafted patients post-allogeneic HSCT with cytopenias of unclear etiology, low counts were found to be associated with T-LGL, especially in patients with prior cytomegalovirus infection, and improved with immunosuppressive therapy (10). The association was found in 47% of the cases, with clonal T-LGL expansion in 22%, oligoclonal in 53%, polyclonal in 4%, and indeterminate in 20% (9). T-LGL expansion was also detected in three patients with R/R non-Hodgkin lymphoma who received chimeric antigen receptor T-cell therapy and experienced prolonged or recurrent cytopenias (11). These clinical scenarios suggest that dysregulation of the immune system, whether suppression or activation of certain cell populations, may trigger T-LGL clonal expansion, which, in turn, may lead to cytopenias. It is not possible for us to know with certainty if the reactive T-LGLs seen in this case were due to teclistamab, but the temporal association is suggestive. With continued administration of immunomodulatory drugs, like bispecific antibodies in patients with MM, more information will be obtained over time regarding T-LGL expansion and concomitant neutropenia.

At the time of detection of T-LGLs, our patient’s restaging PET/CT scan showed FDG uptake concerning for progression of disease in pre-existing lytic lesions. Given the lack of corresponding pain, concurrent biochemical progression of disease, or presence of overt disease in the bone marrow, pseudoprogression was suspected. Although limited, there have been reports of pseudoprogression on imaging in patients with biochemical response to teclistamab (12) and the GPRC5DxCD3-directed bispecific antibody talquetamab (13). Although our patient’s repeat imaging at 3 months after the discontinuation of therapy did not demonstrate complete radiologic remission, there was objective improvement in the FDG uptake of lesions and no new lesions. The patient remained free of new bone pain.

To our knowledge, this is the first reported possible association of T-LGL activation with teclistamab treatment. Mechanistically, teclistamab is designed to work by redirecting T-cells to attack malignant plasma cells (2) and not cause the clonal expansion of T-cells as it occurs in T-LGL leukemia (6). However, the stimulation of the T-cells of the immune system by teclistamab may inadvertently lead to clonal T-LGL expansion. It, thus, may be important to evaluate for pre-existing T-cell abnormalities before initiating therapy to prevent the development of secondary hematologic malignancies.

In conclusion, treatment with teclistamab led to control of the disease in this patient with penta-class refractory MM but had to be discontinued as it appeared to potentially ramp up the patient’s immune system, causing possible reactive measurable changes in the bone marrow T-cell composition that could have led to moderate to severe symptomatic neutropenia. It is suspected that the reactive changes in the setting of the bispecific antibody were also observed simultaneously on PET/CT imaging. High populations of reactive T-LGLs have not been previously reported in the bone marrow of patients receiving T-cell engager therapies, and this may need to be studied further as they may have an impact on the patient clinically or affect their response to treatment radiologically.

## Data Availability

The original contributions presented in the study are included in the article/Supplementary Material. Further inquiries can be directed to the corresponding author.
